# Data augmentation using generative adversarial networks (CycleGAN) to improve generalizability in CT segmentation tasks

**DOI:** 10.1038/s41598-019-52737-x

**Published:** 2019-11-15

**Authors:** Veit Sandfort, Ke Yan, Perry J. Pickhardt, Ronald M. Summers

**Affiliations:** 1 Imaging Biomarkers and Computer-Aided Diagnosis Laboratory, Radiology and Imaging Sciences, National Institutes of Health Clinical Center, Building 10 Room 1C224D MSC 1182, Bethesda, MD 20892-1182 USA; 2 Department of Radiology, University of Wisconsin School of Medicine and Public Health, Madison, WI USA

**Keywords:** Image processing, Diagnostic markers

## Abstract

Labeled medical imaging data is scarce and expensive to generate. To achieve generalizable deep learning models large amounts of data are needed. Standard data augmentation is a method to increase generalizability and is routinely performed. Generative adversarial networks offer a novel method for data augmentation. We evaluate the use of CycleGAN for data augmentation in CT segmentation tasks. Using a large image database we trained a CycleGAN to transform contrast CT images into non-contrast images. We then used the trained CycleGAN to augment our training using these synthetic non-contrast images. We compared the segmentation performance of a U-Net trained on the original dataset compared to a U-Net trained on the combined dataset of original data and synthetic non-contrast images. We further evaluated the U-Net segmentation performance on two separate datasets: The original contrast CT dataset on which segmentations were created and a second dataset from a different hospital containing only non-contrast CTs. We refer to these 2 separate datasets as the in-distribution and out-of-distribution datasets, respectively. We show that in several CT segmentation tasks performance is improved significantly, especially in out-of-distribution (noncontrast CT) data. For example, when training the model with standard augmentation techniques, performance of segmentation of the kidneys on out-of-distribution non-contrast images was dramatically lower than for in-distribution data (Dice score of 0.09 vs. 0.94 for out-of-distribution vs. in-distribution data, respectively, p < 0.001). When the kidney model was trained with CycleGAN augmentation techniques, the out-of-distribution (non-contrast) performance increased dramatically (from a Dice score of 0.09 to 0.66, p < 0.001). Improvements for the liver and spleen were smaller, from 0.86 to 0.89 and 0.65 to 0.69, respectively. We believe this method will be valuable to medical imaging researchers to reduce manual segmentation effort and cost in CT imaging.

## Introduction

Segmentation of organs or pathologies promises to improve medical decision making by adding objective and reliable measurements to the clinical imaging process where this level of quantification would be too time-consuming if done manually.

Convolutional neural networks (CNN) with 2D and 3D inputs have achieved high segmentation performance in various tasks^1^. However, machine learning models currently require large amounts of data, especially if high performance on a diverse dataset is required. Labeling medical image data is a very expensive and time-consuming task. A major issue is that a model trained in a specific dataset may not perform as well when applied in a moderately different real-world dataset (distribution or dataset shift)^2^. In this work, we evaluate the use of generative adversarial networks (GANs) to increase robustness and generalizability of organ segmentation in CT.

There is a strong interest in using unlabeled data to improve deep learning performance. GANs are a very powerful group of networks which can generate plausible new images from unlabeled original images^3^. GANs have been previously used for data augmentation, for example, to generate new training images for classification^4^, to refine synthetic images^5^ or to improve brain segmentation^6^. CycleGANs have also been used to improve segmentation^7–9^.

In many cases, CT scans performed with intravenous injection of iodine contrast agent result in clinically more meaningful and information-rich images, for example by helping to identify or classify tumors. Therefore in many cases, IV iodine contrast-enhanced CT (‘contrast CT’) is preferred over non-contrast CT. Nevertheless, there are many situations where the application of iodine contrast is not feasible due to reduced renal function, contrast allergy, failure of intravenous access during injection or unfavorable risk-benefit ratio (e.g. in certain screening exams like CT colonography). Of note, the change of CT attenuation or image brightness when using IV contrast may be neither uniform nor dependent on the non-contrast CT attenuation of a tissue. The change in CT attenuation depends on various biological and physical factors including the blood flow to a tissue, the amount of extracellular volume of the tissue and the CT technology (e.g. tube voltage of the X-ray tube).

Segmentations of abdominal organs found in public CT datasets are near universally performed on contrast-enhanced CT scans while real-world data contains a certain percentage of non-contrast CT scans. This constitutes a distribution shift - where the training data is different from real-world data - and may adversely affect performance in real-world applications.

We aimed to alleviate this issue by using data augmentation. Using generative adversarial networks (specifically CycleGAN^10^) we generate a synthetic non-contrast version of training data contrast CTs. We then train on the original data while using the synthetic non-contrast CTs for data augmentation.

Rendering images with the appearance of non-contrast CT from original contrast CT data is a non-trivial task. Recently, generative adversarial networks and in this case specifically cycle consistent generative adversarial networks have enabled a true breakthrough in the quality of synthetic image generation^3,10^, reviewed in^11^. The key to this ability is an internal competition between an image transforming network (usually encoder/decoder architecture) and an adversarial network that attempts to discriminate generated synthetic images from real images. In the optimal case, the generated images would be indistinguishable from real images. This technique makes it possible to transform images from one domain (in this case contrast CT) to another domain (in our case non-contrast CT) with *unpaired* images. This task would have been considered by most experts to be impossible to achieve just a few years ago. In the specific type of GAN used, the images are translated back to the original domain to improve consistency, hence the name ‘CycleGAN’.

In the clinical realm, caution is needed. The generated images may *look* like real images, but there is absolutely no assumption that the specific non-contrast images of an actual patient would really be similar to the generated images. Certainly, this is not a magical tool but more a very sophisticated type of ‘style transfer’. In the domain of CT it should be especially emphasized that these images are fundamentally different from what is commonly called ‘virtual non-contrast’ images. Virtual non-contrast images are the product of dual-energy CT scans. This enables a physical/mathematical modeling of the X-ray absorption and generates, within certain limitations, a true measurement of the tissues without the contrast. Of note, in this work, synthetic non-contrast CT images are used for strengthening data augmentation methods but not for actual measurements or diagnostic purposes.

We hypothesize that CycleGAN type data augmentation improves performance in a dataset of non-contrast CT.

## Results

### Synthetic non-contrast CT - qualitative evaluation

**Figure 1:**
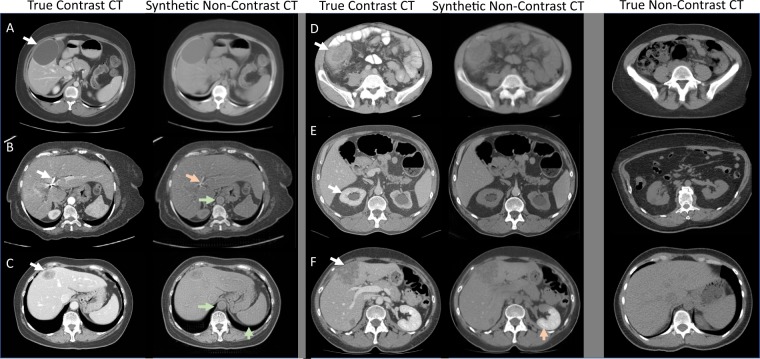
Examples of true IV contrast CT scans (left column) and synthetic non-contrast CT scans generated by a CycleGAN. The rightmost column shows unrelated example non-contrast images. Overall the synthetic non-contrast images appear convincing - even when significant abnormalities are present in the contrast CT scans.

Figure 1 shows typical examples of contrast/synthetic non-contrast pairs where the contrast image is a CT scan which was performed with intravenous contrast agent and the synthetic non-contrast image was generated by the trained CycleGAN. The images also show the performance of the system when faced with various abnormalities/pathologies.

One concern in regard to CycleGAN based contrast to non-contrast transformation is that unusual pathology on the images might not be correctly transformed. Therefore a radiologist screened the CT scans for pathology or difficult anatomy and evaluated the transformed non-contrast images. In the following we will discuss specific details of pathology or difficult anatomy visualized in Fig. 1.

In part A the white arrow indicates a liver cyst with no contrast accumulation. The resulting non-contrast image appears plausible (right panel). In part B the aorta is seen, which is very bright on contrast CT and is correctly reduced in brightness/attenuation on synthetic non-contrast CT (green arrow). In part C the white arrow points to a liver mass which is hypo-attenuating resulting in a plausible synthetic image. Part D shows a colon carcinoma with mild contrast enhancement indicated by the white arrow. This contrast enhancement is correctly reduced in brightness/attenuation in synthetic non-contrast as it would be expected on true non-contrast. In part E the white arrow points to an abnormal kidney with contrast enhancement. On the right panel the brightness/attenuation of the kidney is reduced in a plausible way on synthetic non-contrast CT. But there where also problematic examples where synthetic non-contrast images are not as expected. For example, on image B, indicated by the white arrow, there is a stent present in the biliary system. These stents are marked with radiopaque material and therefore appear very bright on the CT regardless of whether IV contrast is present. In the synthetic non-contrast CT the stent appears much darker (lower attenuation) - this is not expected and incorrect. In part F, while the other features in this image appear plausible on synthetic non-contrast, the kidney still appears as if IV contrast was present in most areas (red arrow), which is incorrect.

In summary, the synthetic non-contrast images appear in most cases to be plausible on quick examination. It should be noted that an experienced radiologist would have no problem discriminating between synthetic non-contrast CT images and actual non-contrast CT images on full resolution images but this may be difficult and take longer on scaled-down images.

### Segmentation results

**Figure 2:**
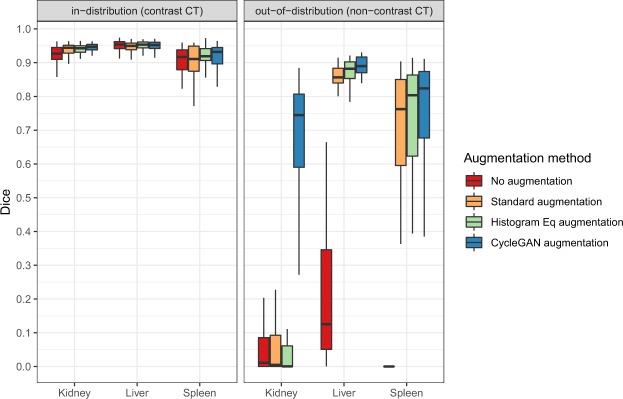
Dice scores of different organs for the tested augmentation methods in the two test sets (in-distribution (contrast CT) vs. out-of-distribution (non-contrast).

Table 1 shows the segmentation performance measured by Dice score of each organ and for the in-distribution (contrast CT) and out-of-distribution (non-contrast) test sets (mean and standard deviation of 5-fold CV). Figure 2 shows box plots for pooled individual segmentation results of 5 cross-validation experiments.

**Table 1:** Segmentation performance measured as Dice score for kidney, liver and spleen. Shown are mean scores and standard deviation of 5 cross-validation experiments.

Organ	Evaluation Dataset*	Augmentation Method			
		None	Standard	Histogram Eq	CycleGAN
Kidney	in-distribution (contrast CT)	0.920 ± 0.013	0.940 ± 0.007	0.939 ± 0.006	**0**.**944** ± **0**.**009**
	out-of-distribution (non-contrast CT)	0.059 ± 0.034	0.090 ± 0.039	0.066 ± 0.027	**0**.**664** ± **0**.**040**
Liver	in-distribution (contrast CT)	0.944 ± 0.005	0.941 ± 0.006	**0**.**948** ± **0**.**003**	0.947 ± 0.003
	out-of-distribution (non-contrast CT)	0.207 ± 0.209	0.860 ± 0.009	0.873 ± 0.015	**0**.**887** ± **0**.**006**
Spleen	in-distribution (contrast CT)	0.884 ± 0.029	0.890 ± 0.037	**0**.**919** ± **0**.**005**	0.904 ± 0.032
	out-of-distribution (non-contrast CT)	0.038 ± 0.009	0.654 ± 0.031	0.648 ± 0.051	**0**.**691** ± **0**.**065**
All Averaged	in-distribution (contrast CT)	0.916	0.924	**0**.**935**	0.932
	out-of-distribution (non-contrast CT)	0.101	0.535	0.529	**0**.**747**

*For definitions see section Experimental Setup. *Mean* ± *sd*.

#### In-distribution performance (contrast CT)

First, in all organs a reasonable baseline performance for standard augmentation segmentation in the in-distribution test set is seen with Dice scores ranging from 0.89 to 0.94. If using no augmentation at all (column ‘None’ in Table 1) the Dice scores were overall similar compared to standard augmentation.

In the in-distribution dataset, CycleGAN augmented results were slightly improved compared to standard augmentation, especially in the spleen images (all p < 0.05).

The histogram equalization augmentation improved segmentation performance compared to standard augmentation in liver and spleen segmentations where it showed better performance than the CycleGAN augmentation.

#### Out-of-distribution performance (non-contrast CT)

In the out-of-distribution non-contrast dataset a near complete loss of performance is seen for the kidney segmentation when using no augmentation, standard augmentation or histogram equalization augmentation with Dice scores of 0.06, 0.09 and 0.07, respectively. When using CycleGAN augmentation a dramatic increase of the Dice score for kidney segmentation is noted (from 0.09 to 0.66, for standard and CycleGAN augmentation, respectively, p < 0.001). Smaller differences but a similar pattern is seen in the liver and spleen segmentations. In all organ tasks, the CycleGAN augmentation showed the best out-of-distribution performance compared with the other augmentation methods.

The out-of-distribution (non-contrast CT) performance when training without any augmentation was greatly reduced compared to standard augmentation as can be seen in the liver and spleen tasks (Dice 0.21 vs 0.86 for no augmentation vs standard augmentation for liver and Dice 0.04 vs. 0.65 for no augmentation vs standard augmentation for spleen).

The histogram equalization augmentation led to a small improvement in mean Dice scores compared to standard augmentation for liver and a small deterioration for kidney and spleen.

### Volume measurement error results

Organ segmentations are frequently used in clinical research for volume measurements. Therefore we calculated the relative volume estimation errors (Methods, Eq. 2). The results for this metric are shown in Table 2. The in-distribution volume measurement errors for CycleGAN augmented segmentations were excellent for kidney and liver (3% and 4%, respectively) and reasonable for spleen (8%). For non-contrast data and in line with the findings on the Dice scores, a striking improvement (reduction) of the volume estimation error is seen for CycleGAN compared to standard augmentation. For example, for the kidney the volume errors were 0.45 vs. 0.19, p < 0.001, and for the liver the volume errors were 0.11 vs. 0.08, p = 0.008, for standard and CycleGAN augmentation, respectively.

**Table 2:** Volume estimation error for kidney, liver and spleen segmentations.

Organ	Evaluation Dataset*	Augmentation Method			
		None	Standard	Histogram Eq	CycleGAN
Kidney	in-distribution (contrast CT)	0.051 ± 0.016	0.038 ± 0.010	0.041 ± 0.0123	**0.032** ± **0.008**
	out-of-distribution (non-contrast CT)	0.334 ± 0.076	0.450 ± 0.126	0.361 ± 0.071	**0.189** ± **0.068**
Liver	in-distribution (contrast CT)	0.047 ± 0.007	0.047 ± 0.008	**0.038** ± **0.004**	0.043 ± 0.010
	out-of-distribution (non-contrast CT)	0.583 ± 0.247	0.107 ± 0.030	0.090 ± 0.026	**0.080** ± **0.022**
Spleen	in-distribution (contrast CT)	0.112 ± 0.014	0.104 ± 0.068	**0.058** ± **0.021**	0.083 ± 0.051
	out-of-distribution (non-contrast CT)	1.487 ± 0.642	0.355 ± 0.0657	0.311 ± 0.060	**0.265** ± **0.094**
All Averaged	in-distribution (contrast CT)	0.070	0.063	0.046	0.053
	out-of-distribution (non-contrast CT)	0.801	0.304	0.254	0.178

Average volume estimation error and standard deviation of 5 cross-validation experiments are shown. Lower volume estimation error indicates higher performance, and bold numbers represent the best result in each line.

In line with the Dice scores findings, histogram equalization augmentation resulted in improved results for in-distribution (contrast CT) segmentations in liver and spleen (best results) while in out-of-distribution (non-contrast CT) the CycleGAN augmentation showed the lowest volume estimation errors.

### Example images

**Figure 3:**
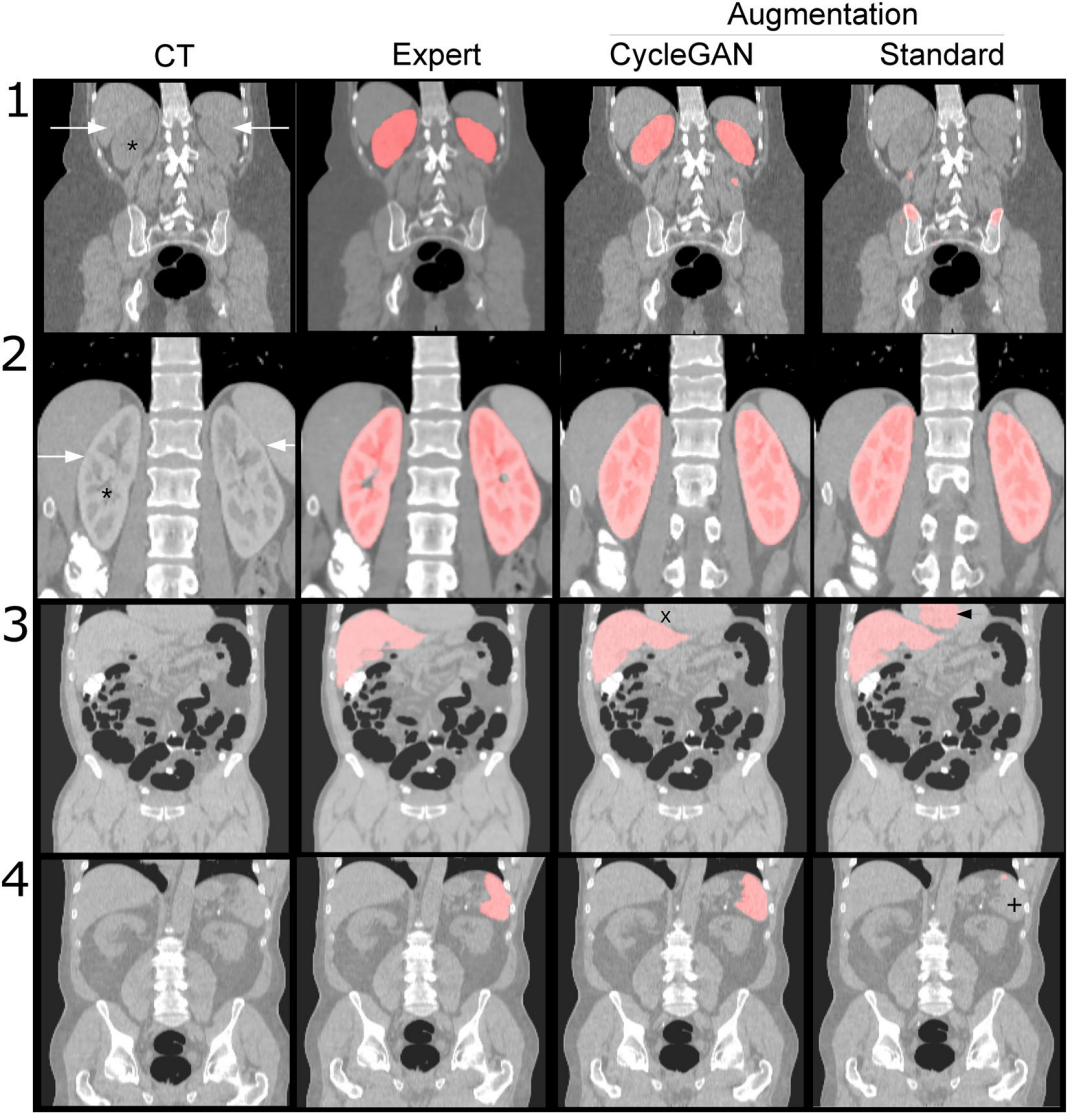
Examples of segmentations. Original CT and expert segmentation are shown in the first and second columns and CycleGAN and standard augmented training results are shown in the third and fourth columns, respectively. For detailed comments see main text.

Figure 3 shows examples of kidney, liver and spleen segmentations. In line with the summary statistics, the segmentations in the in-distribution test set look reasonably good (second row). In the non-contrast kidney example (first row) it becomes clear that the network trained with standard augmentation fails to segment the kidney (right upper image) while the network trained with CycleGAN augmentation gives a relatively good segmentation. In the left column differences between these CT scans can be seen. Due to the high contrast uptake, the kidney appears brighter on contrast images (second row) compared to non-contrast images (first row). This makes the separation of kidney and neighboring organs simpler (white arrows). The contrast agent also results in a specific texture of the kidney which is not seen on non-contrast images (asterisk). In the third row, a liver segmentation on non-contrast images is shown. The boundary between liver and heart is not easily detected in non-contrast CT and the model trained using standard augmentation falsely extends the liver area into the heart area (black arrow, third row, rightmost image). The CycleGAN augmented model correctly respects the liver/heart boundary (marked with x). In the fourth row a spleen segmentation on non-contrast CT is shown. Again it is demonstrated that in a situation with ambiguous boundaries with neighboring structures the CycleGAN augmented segmentation shows a good result while the model trained using standard augmentation fails to detect large parts of the spleen (marked with +).

## Discussion

Deficits in generalization to real-world datasets with moderately different characteristics (distribution-shifts) are major hurdles for the adoption of deep learning methods in clinical imaging.

We hypothesized that by performing data augmentation using generative adversarial networks segmentation performance could be improved in diverse image datasets. We evaluated the use of synthetic non-contrast CT images derived from contrast CT as a data augmentation method.

First, our results showed that in certain tasks, especially kidney segmentation, a model trained on contrast images will fail nearly completely on non-contrast images (Dice scores of 0.94 vs. 0.09 for contrast CT and non-contrast CT, respectively). This is important to recognize, as in the clinical world non-contrast CT scans are frequently performed. Other tasks were less affected, but the pattern was still seen in spleen and liver segmentations. These observations make sense in the context of the iodine content of these organs. Due to the excretion of contrast through the kidney, this organ accumulates contrast agent and therefore the differences between contrast and non-contrast images are large. Intuitively, it is also likely that the U-Net segmentor learns to detect certain typical textures and patterns of kidney tissue caused by the contrast agent which are then not present on the non-contrast scans. This is analogous to a grass detector which learns to detect the color green as an indicator of grass and then fails on black and white images. For a texture comparison see Fig. 3 marked with asterisks.

It should be noted that important pathologies such as tumors also frequently accumulate contrast agents and that a deterioration in performance can be expected if training data does not account for presence or absence of contrast.

Secondly, we observed that augmentation using CycleGAN-generated synthetic images significantly improved segmentation performance in the non-contrast CT test set. Again, the effect was seen strongly in the kidney segmentations (Dice scores of 0.09 vs. 0.66 for standard vs. CycleGAN augmentation). Surprisingly, there was also a trend toward improved segmentation performance in the in-distribution test datasets, especially for the spleen.

Thirdly, histogram equalization augmentation led to improved results compared to standard augmentation for liver and spleen but no improvement for kidney (see Fig. 2). It could be hypothesized that histogram equalization is helpful to some extent to model due to the global increase in brightness or CT attenuation that occurs when performing IV contrast enhanced scans, but it has limitations when there are strong local differences in contrast enhancement within a specific organ such as the kidney, which is the strongest contrast enhancing organ evaluated.

In addition, because volume assessment is an important task in the context of organ segmentation we evaluated the accuracy of volume measurements using relative volume error. These results reemphasized the previous findings with reduction of the measurement error in all examined organs when using CycleGAN based augmentation. We speculate that segmentation performance of many more structures with relevant contrast enhancement may benefit from this augmentation technique.

Methods to leverage CycleGAN in medical images have been described before in the literature. Seeboeck *et al*. used a CycleGAN to adapt between different OCT (optical coherence tomography) retinal scanners^7^. This approach differs from our work in that the model is trained on images from one type of scanner and then a CycleGAN attempts to make the testing scans from another scanner to be more similar to the training scans. In our work, we used the CycleGAN to train a U-Net that is capable of segmenting scans from both domains. In the case of contrast this may be more useful because it is not always known if a scan was performed with or without contrast and there is a large continuous range of contrast doses. Zhang *et al*. have used a complex 3D Cycle-GAN with an additional shape-consistency loss to enable modality transfer between cardiac MRI and cardiac CT by incorporating a subset of labeled data in both modalities^8^. This method is able to significantly increase the performance of segmentations but it requires labels in both domains. Huo *et al*. have proposed a sophisticated cross-modality segmentation network which does not need labels in the target domain^9^. They explored the task of transferring labels from MRI to CT images with very good results. Our work focused on the issue of contrast and non-contrast CT which are not usually perceived as distinct modalities. However, given the large differences in performance shown in Fig. 2, in the context of CNNs they probably should be considered to be different modalities. Our approach has the advantage that the synthetic training data can be inspected and evaluated for problematic cases and errors which may be helpful in a clinical scenario where interpretability is important. In addition a major difference is that we were able to perform the segmentation step in 3D.

A limitation of our method is that the CycleGAN method is applied to single slices (2D) of the 3D input volume. This leads to slice-to-slice inconsistencies which may adversely affect performance. This problem would be best alleviated by a fully 3D CycleGAN, which is challenging due to GPU memory considerations. In addition, there are limitations within the CycleGAN method itself. The relationship between contrast to non-contrast CT is basically many-to-one (as multiple contrast phases or intensities would still correspond to the same non-contrast image). Within the framework of CycleGAN this leads to a one-to-many relationship in the reverse transformation, and there are difficulties with this type of transformation. Novel modifications of the idea behind CycleGAN will likely solve this issue, for example the concept of augmented CycleGAN^12^. This concept would also enable generation of multiple contrast variants from a synthetic or real non-contrast image, such as different contrast intensities and phases that could further enhance data augmentation.

In summary, our findings show that generative adversarial networks are a very useful augmentation tool for CT image segmentation. Given the scarcity and cost of labeled data, all means should be used to make more efficient use of the available data. Augmentation using spatial transformations is standard and best practice but in CT images complex modification of attenuation values is not typically performed. We present a relatively simple method that can improve segmentation performance in a variety of scenarios in CT imaging.

## Methods

### Data

Data for the in-distribution dataset (contrast CT) were obtained from the following sources:

Kidney: NIH Pancreas-CT dataset (unlabeled images available on TCIA, The Cancer Imaging Archive), Liver and Spleen: Data Decathlon data set^13^. The number and dimensions of images are shown in Table 3. Image data for the out-of-distribution (non-contrast CT) data set were obtained from a non-public screening study^14^ and were acquired at a different hospital and for a different indication (virtual colonoscopy). Test set labels of liver, kidney and spleen segmentations (n = 10) were generated by a physician with >5 years of medical imaging experience using Slicer3D. For the CycleGAN-training images from the DeepLesion data set^15^ were used.

**Table 3:** Numbers of images in each dataset.

Dataset	N in-Distribution Total	Train/Val/Test	N Out-of-Distribution Test	Typical Dimensions
Kidney NIH	66	50/3/13	10	512 × 512 × 220
Liver DataDecathlon	231	179/9/43	10	512 × 512 × 500
Spleen DataDecathlon	40	30/2/8	10	512 × 512 × 90

### Experimental setup

**Figure 4:**
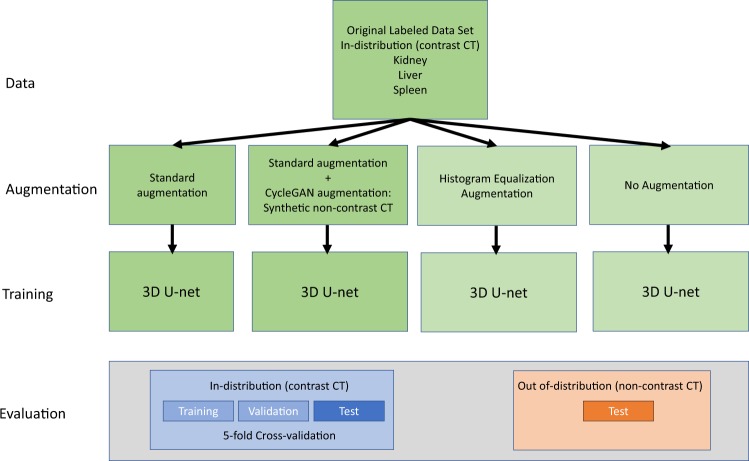
Overview of the experimental setup.

An overview of the experimental setup is shown in Fig. 4. The pre-specified aim was to compare segmentation performance of a 3D U-Net when trained using standard augmentation vs. CycleGAN +standard augmentation. In standard augmentation flips, rotation, non-rigid deformation and crop were applied. In CycleGAN augmentation, in addition, either the original image was used or a synthetic non-contrast CT image was generated/transformed from the contrast CT using a CycleGAN (probability 0.5 for each). In addition to these pre-specified analyses, we also performed explanatory analyses with no augmentation at all and with histogram equalization augmented training. No significance testing was performed for these post-hoc analyses.

For the in-distribution dataset the train/validation/test split was performed with a relation of 75%/5%/20%. We chose the relatively low amount of validation data to maximize the amount of data available for training, but on the other hand this may introduce bias in some scenarios. There was one 3D volume per patient, therefore, no cross-contamination of test data occurred.

For the in-distribution (contrast CT) dataset classic 5-fold cross-validation was used. The out-of-distribution (non-contrast CT) data was not part of the training data, therefore classic cross-validation was not feasible. To gather the variability of training on different data folds we evaluated the complete test dataset with each fold of the evaluation for the out-of-distribution data. Dice scores and volume estimation errors were compared.

### Neural network architecture and training

**Figure 5:**
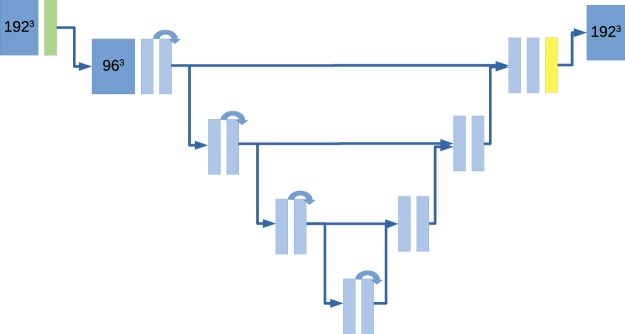
Basic architecture of the U-Net used. We inserted a strided convolution (green) as the first layer (stride 2) with a large kernel (7 × 7 × 7). This modification is complemented by a transposed convolution in the last layer (yellow). This reduces greatly the need for feature map memory and significantly increases the maximum input size. Curved arrows denote residual connections. Note that there is no skip connection at the highest level.

For segmentation a modified 3D U-Net^16^ with residual connections was implemented in PyTorch, inspired by^17,18^, see also Fig. 5. For each organ a separate model was trained. We used leaky ReLU as the activation function and replaced batch normalization with group normalization (group size 16) because it was shown to result in improved performance in the setting of low batch size^19^. In addition, to enable processing of larger input volumes we inserted a strided convolution (stride 2, kernel size 7) after the input layer while adding a corresponding transposed convolution layer for learned up-sampling as the final layer. While theoretically computationally more expensive compared with multiple smaller kernel convolutions this approach drastically reduces the amount of feature map memory needed in the first layers compared to a classical U-Net of the same size. These adaptations enabled processing of clinically acceptable input volume sizes of up to 256 × 256 × 192 on commercially available large-memory GPUs. Experiments were performed with 192 × 192 × 192 volumes to keep training times amenable to a shared HPC environment (<10 hours). A Dice loss function was used (Eq. 1, where s = 1 for training and s = 0 for evaluation).

1 $$Loss=1-2\frac{|{X}_{i}\cap {Y}_{i}|+s}{|{X}_{i}|\cup |{Y}_{i}|+s}$$

Training was performed on the NIH Biowulf cluster using 2-GPU nodes (2xNVIDIA K80, for a total of 4 logical GPUs with 12 GB each) with a batch size of 4 (pytorch nn.DataParallel). The model consumed about 5-6 GB of GPU memory per logical GPU during training. Training was stopped after 10,000 iterations or when no improvement in the validation set occurred for 10 epochs. The model with the best validation performance was used for further testing.

### Augmentation methods

#### Generation of synthetic non-contrast CT images using CycleGAN

For the training of the CycleGAN we manually selected contrast (n = 136) and non-contrast CTs (n = 70) from a superset of the DeepLesion NIH data set^15^ (complete and uncropped CTs used to generate the DeepLesion collection). These data were used to train a ResNet classifier to distinguish contrast and non-contrast CTs. Using this classifier all CTs in the DeepLesion dataset were classified into contrast and non-contrast CT groups. We only used CTs where the probability for being a contrast CT was >0.8 or <0.2. Many of the non-contrast scans were low-dose and had excessive noise causing the artificial introduction of noise in the generated images. Therefore we only included non-contrast CTs with a noise measured by standard deviation of fat of <15 HU. This resulted in 10,681 contrast CTs and 603 non-contrast CTs available for the training of the GAN. Of note, no segmentation labels are available for this data set. The publicly available implementation of CycleGAN was used^10^. For input images, CT attenuation numbers were clipped at −200 and 300 HU before normalization, as this is a range where iodine contrast affects the attenuation the most. Resolution was 256 × 256 and training was performed for 3 million iterations (3 GPUs, batch size 6). Inference results were randomly sampled and checked by an imaging physician for plausibility.

#### Histogram equalization augmentation

As an additional comparison, we performed data augmentation using histogram equalization^20^ to shift the histogram of contrast CTs toward a non-contrast CT histogram using a Python implementation of the MATLAB function histeq. The models were trained using a 0.5 probability for the original image and the histogram equalized image. Standard augmentation was used in addition. Training and evaluation were also repeated with no data augmentation. The advantage compared to CycleGAN augmentation is that histogram equalization is well understood and does not show any unpredictable ‘black-box’ behavior.

#### Standard data augmentation

A typical 3D data augmentation pipeline was used in all experiments including flipping, random crop, 3D rotation (up to 30), and elastic 3D deformation (b-spline transformation, 10 control points, deformation Gaussian *σ* = 8). In addition, in experiments with enabled CycleGAN augmentation, the precomputed synthetic non-contrast CT images were used instead of the original CT data with a probability of 0.5. All data were normalized to zero mean and unit variance. To improve training times with complex on-the-fly augmentations on multi-GPU machines, we cached augmented data and used 16 variants for each volume (where each variant is the result of applying all above-mentioned augmentation methods).

### Statistical analysis

Test datasets (in-distribution test dataset and non-contrast test dataset) were evaluated on Dice loss and volume error. We decided to add a volume error metric because an important use case for organ segmentations is volume assessment. We calculated the relative volume estimation error as:

2 $$Erro{r}_{Volume}=|\frac{Volum{e}_{ExpertSegmentation}-Volum{e}_{UNetSegmentation}}{Volum{e}_{ExpertSegmentation}}|.$$

For statistical testing, the Wilcoxon signed-rank test was computed for individually paired samples.

## Data Availability

The datasets analyzed during the current study are available in the Medical Data Decathlon repository on medicaldecathlon.com and TCIA: wiki.cancerimagingarchive.net/display/Public/Pancreas-CT.
